# Digging deep into cells to find mechanisms of kidney protection by SGLT2 inhibitors

**DOI:** 10.1172/JCI167700

**Published:** 2023-03-01

**Authors:** Katherine R. Tuttle

**Affiliations:** 1 Providence Medical Research Center, Providence Inland Northwest Health, Spokane, Washington, USA.; 2 Division of Nephrology, University of Washington School of Medicine, Seattle, Washington, USA.

## Abstract

The sodium-glucose cotransporter-2 (SGLT2) is expressed on the luminal side of proximal tubule epithelial cells in the kidney. While pharmacological inhibition of SGLT2 provides kidney protection in diabetic kidney disease (DKD), the molecular mechanisms remain unclear. In this issue of the *JCI*, Schaub et al. report on the changes in single-cell transcriptional profiles of young participants with type 2 diabetes who received SGLT2 inhibitors. Treatment with SGLT2 inhibitors restored metabolic perturbations in proximal tubular cells and reduced expression of the inflammatory signaling molecule mTORC1. Notably, changes in transcripts and mTORC1 were also found in the kidney of a diabetes mouse model treated with an SGLT2 inhibitor, supporting use of this model for further studies. These findings reveal cellular mechanisms of SGLT2 inhibitors and are important for advancing therapeutic targets in the treatment of DKD.

## A history of sodium glucose cotransporter inhibitors

**Figure 1:**
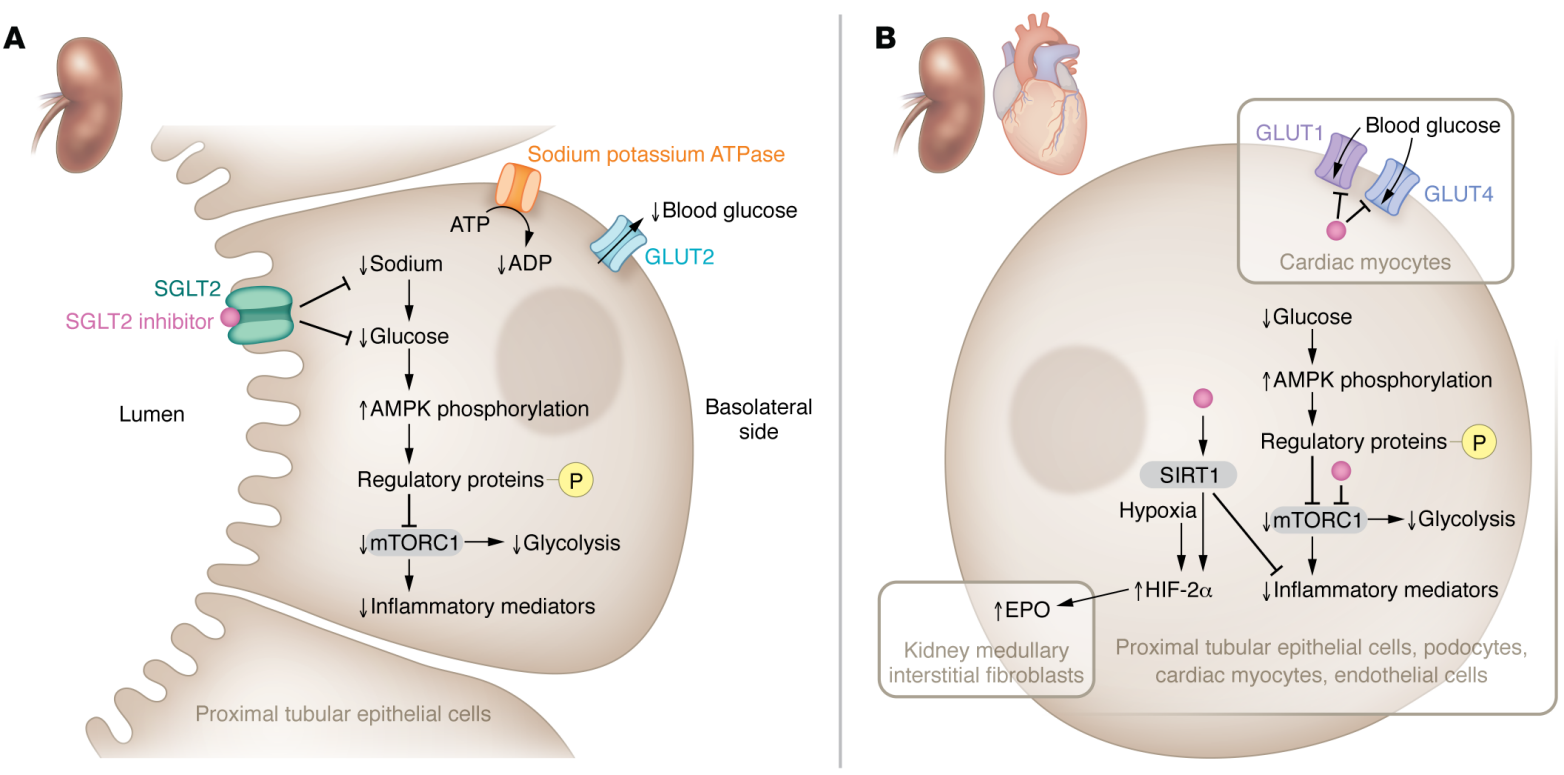
SGLT2 inhibitors mediate kidney-protective effects via receptor- and non-receptor-mediated pathways. (**A**) SGLT2 inhibitors regulate inflammation and glycolysis through SGLT2 receptors. The binding of an SGLT2 inhibitor to the SGLT2 receptor blocks glucose reabsorption from the luminal side of proximal tubular epithelial cells in the kidney, leading to low intracellular glucose, and consequently, less glucose transport across the basolateral side into the blood by GLUT2 (4). Reduced sodium reabsorption at this site also lessens ATPase activity and the conversion of ATP to ADP, which generates energy for solute transport. Further, low intracellular glucose activates AMPK, which phosphorylates regulatory proteins that inhibit mTORC1 signaling (21). Importantly, suppression of mTORC1 may block stimulatory actions to promote expression of inflammatory mediators and glycolysis at this site. (**B**) SGLT2 inhibitors may also modulate inflammation, intracellular glucose levels, and EPO production through non-receptor-mediated pathways. In a variety of cells from the kidney (e.g., proximal tubular epithelial cells, podocytes, fibroblasts) and the cardiovascular system (e.g., cardiac myocytes, endothelial cells), SGLT2 inhibitors may have off-target effects. SGLT2 inhibitors can putatively bind mTORC1 intracellularly to inhibit expression of inflammatory mediators and/or glycolysis depending on cell type (19). Cardiac myocytes contain GLUT1 and GLUT4 that can be blocked by intracellular interaction with SGLT2 inhibitors and, consequently, lower glucose transfer into the cell along with AMPK activation that may also inhibit mTORC1 (20). Conversely, SGLT2 inhibitors can dock with SIRT1 and increase its activity to oppose expression of inflammatory mediators (22, 23). In kidney medullary interstitial fibroblasts, SIRT1 and hypoxia could activate HIF-2α and thereby increase EPO production (25).

The concept of using sodium-glucose cotransporter-2 (SGLT2) inhibitors for treatment of kidney disease has been a story of remarkable serendipity. In a sense, it has been a story line of science in reverse — clinical observations driving preclinical research — a turnaround for the usual order of inquiry for unraveling potential therapeutic mechanisms. The concept of inhibiting SGLTs in the kidney tubules to induce glucosuria, and thereby lower blood glucose and correct insulin resistance, was introduced by Rossetti, DeFronzo, and colleagues in a seminal study of diabetic rats published in the *JCI* in 1987 (1). They studied an oral agent, phlorizin, but it was not feasible for clinical translation because of breakdown in the gastrointestinal tract and inhibition of gut SGLT1, leading to diarrhea. Cloning and characterization of SGLTs in the human body soon followed (2, 3). SGLT2 was found on the luminal side of proximal tubule epithelial cells (Figure 1A) and not elsewhere, which made this receptor an ideal target for pharmacologic inhibition (3, 4). Glucose reabsorption has high energy requirements due to coupling of ATPase with sodium reabsorption (4). Glucose moves across a concentration gradient in proximal tubular epithelial cells into the blood by facilitated transport via glucose transporter 2 (GLUT2) on the basolateral side. SGLT2 inhibitors with proven benefit for kidney protection (canagliflozin, dapagliflozin, and empagliflozin) are aryl-C-glucosides that are not cleaved in the gut with high selectivity for SGLT2 over SGLT1 (5–7). Notably, about 90% of the filtered glucose load is reabsorbed in the proximal tubular S1 and S2 segments (4). Although SGLT2 inhibitors block glucose reabsorption in the S1 and S2 segments, increased glucose reuptake by SGLT1 in the proximal tubular S3 segment results in a net inhibitory effect of about 50% of the filtered glucose load.

As drug development moved forward for hyperglycemia, cardiovascular outcomes trials (CVOTs) for safety after regulatory approval of glucose-lowering agents were required by the US Food and Drug Administration starting in 2008 (8). The EMPA-REG OUTCOME trial was the first CVOT to demonstrate efficacy as well as safety of an SGLT2 inhibitor for major adverse cardiovascular events in type 2 diabetes. It was also the first to show benefits of a glucose-lowering agent for protection against a range of secondary kidney disease end points: albuminuria onset or progression to macroalbuminuria, doubling of serum creatinine with estimated glomerular filtration rate (eGFR) of less than 45 mL/min/1.73 m^2^, kidney failure, and death due to kidney disease (9). Similar results on secondary kidney disease end points were subsequently demonstrated with canagliflozin, dapagliflozin, and ertugliflozin in their respective CVOTs (10–12). These benefits were verified by a trilogy of trials with kidney disease end points as the primary outcomes: CREDENCE, DAPA-CKD, and EMPA-KIDNEY (5–7). Together, these trials, meta-analyzed with the CVOTs and heart failure trials, found clear superiority of SGLT2 inhibitors compared with placebo, with a relative risk reduction of 40% for kidney disease progression in patients with or without type 2 diabetes (13).

## Mechanisms of kidney protection by SGLT2 inhibitors

Despite the palpable excitement over SGLT2 inhibitors as a breakthrough therapy for kidney disease, a paucity of preclinical studies has been available to elucidate the underlying mechanisms. A clue came from the clinical trials themselves based on consistent observations of an early, within days, eGFR dip with subsequent flattening of the slope for eGFR decline, culminating in preservation of kidney function over time (14). A series of studies in humans, as well as experimental mouse and rat models, pointed to reduction in glomerular hyperfiltration as a key hemodynamic mechanism of kidney protection by SGLT2 inhibitors (15–17). A leading conceptual model proposes that these drugs restore tubuloglomerular feedback by increasing distal tubular solute delivery and consequent sodium reabsorption in the macula densa segment of the distal convoluted tubule. When this energy-requiring process uses ATPase for sodium reabsorption, it generates adenosine, a vasoactive paracrine factor that promotes glomerular afferent arteriolar constriction and possibly efferent arteriolar dilation (17). The resulting hemodynamic changes are posited to blunt glomerular hyperfiltration by reducing glomerular hyperperfusion and hypertension. However, other potential pathways for kidney protection remain largely unexplored.

## Receptor-mediated SGLT2 inhibition may shift energy requirements

In this issue of the *JCI*, Schaub et al. describe changes in single-cell transcriptomics among young people with type 2 diabetes who were SGLT2 inhibitor users compared with nonusers and healthy controls (18). Although the comparison groups were small (*n* = 6–10 each) and SGLT2 inhibitors were prescribed for clinical indications, the study observations provide detailed molecular information at the cellular level in the human kidney according to diabetes status and SGLT2 inhibitor use. Many of the structural features of early diabetic kidney disease (DKD) were diminished in the group treated with SGLT2 inhibitors along with cellular transcriptional profiles across nephron segments, reflecting restoration of metabolic perturbations toward normal. Overall, evidence of less glycolysis, gluconeogenesis, and tricarboxylic acid cycle activity was observed in proximal tubular cells, while the reverse was true in cells from distal segments, such as the medullary thick ascending limb. In all nephron segments, less expression of mTORC1, an inflammatory signaling mediator induced by metabolic and nutrient stressors, and phosphorylated S6 protein, an mTORC1 activity marker, was noted with SGLT2 inhibitor treatment. Corresponding changes in transcripts and mTORC1 were found in the kidney cortex from a mouse model of type 2 diabetes treated with an SGLT2 inhibitor, supporting use of this model for further studies. Transcriptomic changes for processes related to cellular energetics (e.g., lower glycolysis, gluconeogenesis, and tricarboxylic acid cycle activity) in the proximal tubule and the opposite in the medullary thick ascending limb could be a consequence of shifts in energy requirements related to solute transport under receptor-mediated SGLT2 inhibition (Figure 1A).

## Putative non-receptor-mediated effects of SGLT2 inhibition

Not all of the Schaub et al. study findings fit neatly within the paradigm of receptor-mediated SGLT2 inhibition (18). Suppression of mTORC1 by lowered intracellular glucose and activation of AMPK may also reduce glycolysis (19). Decreases in readouts for the mTORC1 pathway were observed throughout nephron segments, effectively uncoupling these changes from solute transport (18). Importantly, SGLT2 inhibition engenders relative cell starvation (Figure 1B), as opposed to a nutrient overload state, which may occur in a variety of cell types by non–SGLT2 receptor mechanisms (20). Within cardiac myocytes, empagliflozin can bind and block GLUT1 and GLUT4 intracellularly, thus reducing glucose transport into cells (21). Low intracellular glucose, in turn, may activate AMPK and inhibit mTORC1 signaling via phosphorylation of upstream regulatory proteins (19, 21). Additionally, SGLT2 inhibitors may directly interact with mTORC1 or sirtuin 1 (SIRT1) to reduce mTORC1 activity and inflammatory signaling within various heart and kidney cells (22, 23). Interestingly, the increase in hemoglobin seen with SGLT2 inhibition is associated with a rise in erythropoietin (EPO) (24). However, EPO is made by kidney medullary interstitial fibroblasts rather than the tubular epithelium (25). Direct SIRT1 induction by docking with SGLT2 inhibitors in fibroblast cells or indirect activation from interstitial hypoxia in the medulla could increase EPO production by stimulation of HIF-2α (20, 25). Finally, the SGLT2 inhibitor–related increase in transcription of metallothionein, a mitigator of damage from oxidative stress, across most proximal and distal tubular segments is a finding that warrants exploration.

## Translational implications and conclusions

These important observations offer insights into how SGLT2 inhibitors may protect the kidney that take the evidence beyond investigations of nephron solute transport and glomerular hemodynamics to identification of cellular mechanisms. From a translational perspective, such data are vital for improving therapeutic targeting of SGLT2 inhibitors and for developing even more successful approaches. Nevertheless, several limitations are worthy of consideration for contextualizing the study findings. Although participants were high-risk patients with type 2 diabetes by demography (e.g., youth) and risk factors (e.g., obesity), they had little to no clinically manifest DKD at the time of the kidney biopsy. Therefore, it is unclear which of them will actually progress to DKD or what features may predict progressors versus nonprogressors. Confounding by indication is also a concern particularly with the small observational case-control study design. Notably, patients who received SGLT2 inhibitors could conceivably be either higher or lower risk for DKD based upon clinical decisions made outside the study. Although the SGLT2 inhibitors appear to have a class effect for protecting the kidney that is largely dose independent, biases may also exist due to use of various SGLT2 inhibitors in different doses. Other kidney-protective agents (e.g., angiotensin-converting enzyme inhibitors or angiotensin-receptor blockers) were also used more commonly in the SGLT2 inhibitor users (30%) than in nonusers (17%) (18).

In conclusion, investigations linking deep phenotyping of humans, including kidney tissue–based interrogation by advanced ‘omics and rapidly evolving methodologies, are critical for unraveling fundamental mechanisms of disease in this structurally, functionally, metabolically, and immunologically complex organ. Discovery of how SGLT2 inhibitors have unlocked previously unimagined kidney protection is an important step toward advancing therapeutic targets to meet the enormous unmet need for better treatment of patients with DKD.

## Version 1 03/01/2023

Electronic publication
